# BAC-HAPPY Mapping (BAP Mapping): A New and Efficient Protocol for Physical Mapping

**DOI:** 10.1371/journal.pone.0009089

**Published:** 2010-02-08

**Authors:** Giang T. H. Vu, Paul H. Dear, Peter D. S. Caligari, Mike J. Wilkinson

**Affiliations:** 1 Institute of Biological, Environmental and Rural Sciences, Aberystwyth University, Aberystwyth, United Kingdom; 2 Medical Research Council Laboratory of Molecular Biology, Cambridge, United Kingdom; 3 Sumatra Bioscience, Singapore, Singapore; 4 BioHybrids International, Woodley, United Kingdom; New England Biolabs, Inc, United States of America

## Abstract

Physical and linkage mapping underpin efforts to sequence and characterize the genomes of eukaryotic organisms by providing a skeleton framework for whole genome assembly. Hitherto, linkage and physical “contig” maps were generated independently prior to merging. Here, we develop a new and easy method, BAC HAPPY MAPPING (BAP mapping), that utilizes BAC library pools as a HAPPY mapping panel together with an Mbp-sized DNA panel to integrate the linkage and physical mapping efforts into one pipeline. Using *Arabidopsis thaliana* as an exemplar, a set of 40 Sequence Tagged Site (STS) markers spanning ∼10% of chromosome 4 were simultaneously assembled onto a BAP map compiled using both a series of BAC pools each comprising 0.7x genome coverage and dilute (0.7x genome) samples of sheared genomic DNA. The resultant BAP map overcomes the need for polymorphic loci to separate genetic loci by recombination and allows physical mapping in segments of suppressed recombination that are difficult to analyze using traditional mapping techniques. Even virtual “BAC-HAPPY-mapping” to convert BAC landing data into BAC linkage contigs is possible.

## Introduction

The emergence of high-throughput, so-called next-generation sequencing technologies (http://www.454.com; http://www.appliedbiosystems.com; http://www.illumina.com; http://www.helicosbio.com; http://www.pacificbiosciences.com) heightens demand for fast and low-cost strategies to merge linkage and physical maps to accelerate genome sequencing and refine comparative mapping projects [1], [2]. The ideal scenario would be to merge genetic linkage and physical mapping efforts into a single pipeline for sequence assembly and to order contigs within linkage groups.

At present, genome sequencing projects typically apply “BAC by BAC” approaches [3], [4] (http://sgn.cornell.edu/about/tomato_project_overview.pl) and/or whole genome shotgun sequencing [5] (http://www.genome.gov; http://www.potatogenome.net/; http://www.brachypodium.org) to secure consensus sequence contigs and eventually whole genome sequences. For BAC by BAC sequencing, tiling BACs into contiguous linear arrays is usually achieved by high throughput fingerprinting [6], [7]. The minimum number of overlapping BACs is sought for genome-wide sequencing coverage. As work progresses, BACs will initially assemble into short but numerous contigs. For a simple genome and given sufficient time and resources, these contigs should eventually merge to form a single contig per chromosome. In practice this outcome is prevented by numerous stretches of repetitive DNA, cloning biases and the sheer size of larger genomes. To illustrate, the barley physical mapping project proposes to fingerprint 550,000 clones (12–15x coverage of the 5500 Mbp barley genome) of 7 libraries that were constructed using 4 different restriction enzymes and including also a random sheared library. So far 176,000 BAC clones (∼4x coverage) have been fingerprinted and assembled into 26,000 contigs, which on average contained only 5 clones each, and many of which are singletons consisting of a single BAC (www.barleygenome.org). Under such circumstances, the prime objective is to arrange the contigs into an order that accurately reflects their position along the respective linkage group.

Assembly of the genome sequence is facilitated by paired end sequencing to generate larger scaffolds [2], [8]–[10]. However, the high-resolution genetic and physical maps are still required as a skeleton for sequence assembly [1], [11], [12]. There are two main limitations to generating a genome map: (i) Genetic linkage mapping obligatorily depends on access to polymorphic loci that segregate within the mapping population(s) and (ii) mapping is hampered by unequal distribution of recombination events along the genome. In particular, there are regions of suppressed recombination around centromeres and other heterochromatin-rich regions of large genomes that impair congruence of genetic linkage mapping and genomic distance [13]. In consequence there is only modest correlation between genetic linkage (based on recombination frequency) and physical distance, especially within large genomes (e.g. [13]). Shotgun whole genome sequencing usually provides a reasonable number of contigs harbouring most of the genes. However, a reliable and robust strategy to assemble these contigs into an accurate order that reflects the reality of the genome sequence is still lacking [1].

Here we describe a new procedure called BAC HAPPY MAPPING (BAP mapping) that is designed to overcome these limitations. BAP mapping combines the principles of the BAC landing method [14] with HAPPY mapping [15], [16]. Conventional HAPPY (HAPloid DNA samples analysed using the PolYmerase chain reaction) mapping uses panels of sheared genomic DNA diluted to 0.7x haploid genome equivalents and generates a map based on the premise that linked pairs of single copy STS markers (HAPPY markers) will co-segregate significantly more frequently than unlinked markers and in a manner that is proportional to their physical proximity [15]–[19]. BAP mapping exploits the same principle but creates sub-genome DNA templates by mixing BACs to form partial coverage three-dimensional (3D) BAC pools. This simple pooling strategy provides the most direct means to derive colony coordinates (plate ID, row ID and column ID) for BAC landing applications. Other pooling strategies (such as 2D, [20], and DNA Sodoku, [21]) have been raised for multiplexed high-throughput sequencing but are less favourable for BAC library screening or anchoring purposes because they require a higher number of pools to be screened for each marker. The sub-genome BAC pools generated by the 3D pooling strategy also have the advantage of being replicable and, because insert sizes are in the 50–200 kb size range, provide high resolution and eventually result in a BAC tiling path. The subsequent use of conventional high molecular weight (HMW) sheared genomic DNA creates a long range HAPPY panel (Mbp-sized DNA molecules) that links the shorter contigs obtained from the BAC panel into chromosome-wide contigs.

Here, we used *A. thaliana* as an exemplar to validate the BAP mapping approach; a linkage map and a BAC tiling path of a 1.8 Mbp region of chromosome 4 were created. This approach merges for the first time genetic and physical mapping in one pipeline and generates a “genomic” map in an easy and fast procedure at low cost. Our approach bears the potential for universal applications and will play an important role in sequencing, mapping, and assembly of many large genomes for biomedical and agricultural purposes.

## Results

### Rationale of the BAP Mapping Method

**Figure 1:**
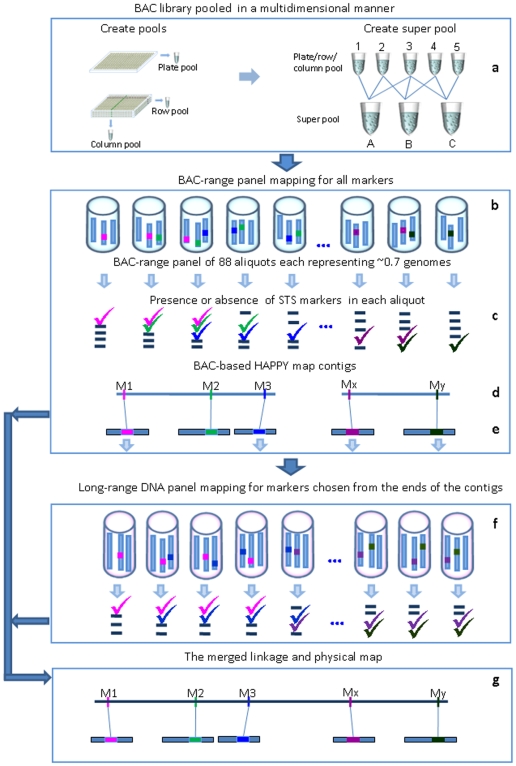
Overview of the BAP mapping method. (a) A genomic BAC library is pooled in a 3-dimensional fashion (1^st^ D for plate ID; pool all unique clones for each plate and create overlapping superpools of plate pools; 2^nd^ D for row ID: pool all clones in identical rows over several plates [3.5 plates for *Arabidopsis*] and create overlapping ‘superpools’ of row pools to allow row identification; 3^rd^ D pool for column ID: pool all clones in identical columns over several plates [3.5 plates for *Arabidopsis*] and create overlapping ‘superpools’ of column pools to allow column identification) to generate the BAC-range panel. (b) Of 96 wells of a plate, 88 contain random aliquots of a BAC library (8 wells are reserved for controls), each BAC aliquot contains DNA corresponding 0.6–0.7 fold coverage of the genome. The presence of single copy STS markers (M1-My) is indicated by various colours in the aliquots. (c) The presence of markers found after MT-PCR-HRM. (d) The BAC range panel mapping results allow creation of short linkage maps, and (e) at the same time, to establish a corresponding BAC tiling path. (f) To link the shorter contigs obtained from the BAC panel and to close the gaps between contigs, markers chosen from the ends of the contigs are mapped by the long-range (large size) DNA panel. (g) The merged linkage and physical map as the final result.

The principle of the BAP mapping method is illustrated in Figure 1.

The BAP map is generated in two steps: the first uses a panel created by mixing BAC clones; the second step uses a long range panel comprised of genomic DNA. For the first step, a genomic BAC library is pooled in a three-dimensional manner (Figure 1a) to create BAC-range-panel (Figure 1b; 88 BAC pools plus 8 wells for controls). It is important for HAPPY mapping [15], that the BAC aliquots contain less than one haploid genome (0.6–0.7 fold coverage of the genome). Based on the known size of the investigated genome (G kb) and on the average insert size of the BAC library, it is possible to calculate the optimal DNA content in the aliquots according to the formula n = ([G/X]*0.7), where n is the number of BAC clones needed to be pooled in an aliquot to contain 0.7 fold genome coverage. Such a BAC-range panel has the advantage that the locations of the clones themselves automatically emerge during the mapping process, creating simultaneously a linkage map (Figure 1d), a strict physical map with precise order of and distance between markers (Figure 1e), and a BAC tiling path.

The BAC-range panel contains DNA fragments of a mean size of ∼100 Kbp (typically in the range 80–150 Kbp) and allows detection of linkage between markers up to ∼80 Kbp apart and so provides fine resolution ordering of STS markers. At the same time, the relatively small size of the BAC clones also is a shortcoming of maps created from this panel: the short span of each member within the pool limits its scope to traverse gaps (>100 Kbp) caused by incomplete marker coverage, by marker clustering, or through the presence of large repetitive regions of DNA (Figure 1d, e). It is to address this point that a second HAPPY mapping panel is created (Figure 1f), this time constructed using HMW fragments of genomic DNA. Whilst the map created from this panel will tend to lack fine resolution, its primary purpose is to map contigs generated by the BAC-range HAPPY/Physical map into order, thereby closing the gaps and yielding both fine resolution and integrative coverage. In order to execute this strategy, all markers are mapped on the BAC-range panel to obtain maximum resolution and a BAC tilling path; then, only a subset of markers from contig ends will be mapped on the long-range panel to link contigs obtained from the BAC panel and to close the gaps between wider spaced markers (Figure 1g).

**Figure 2:**
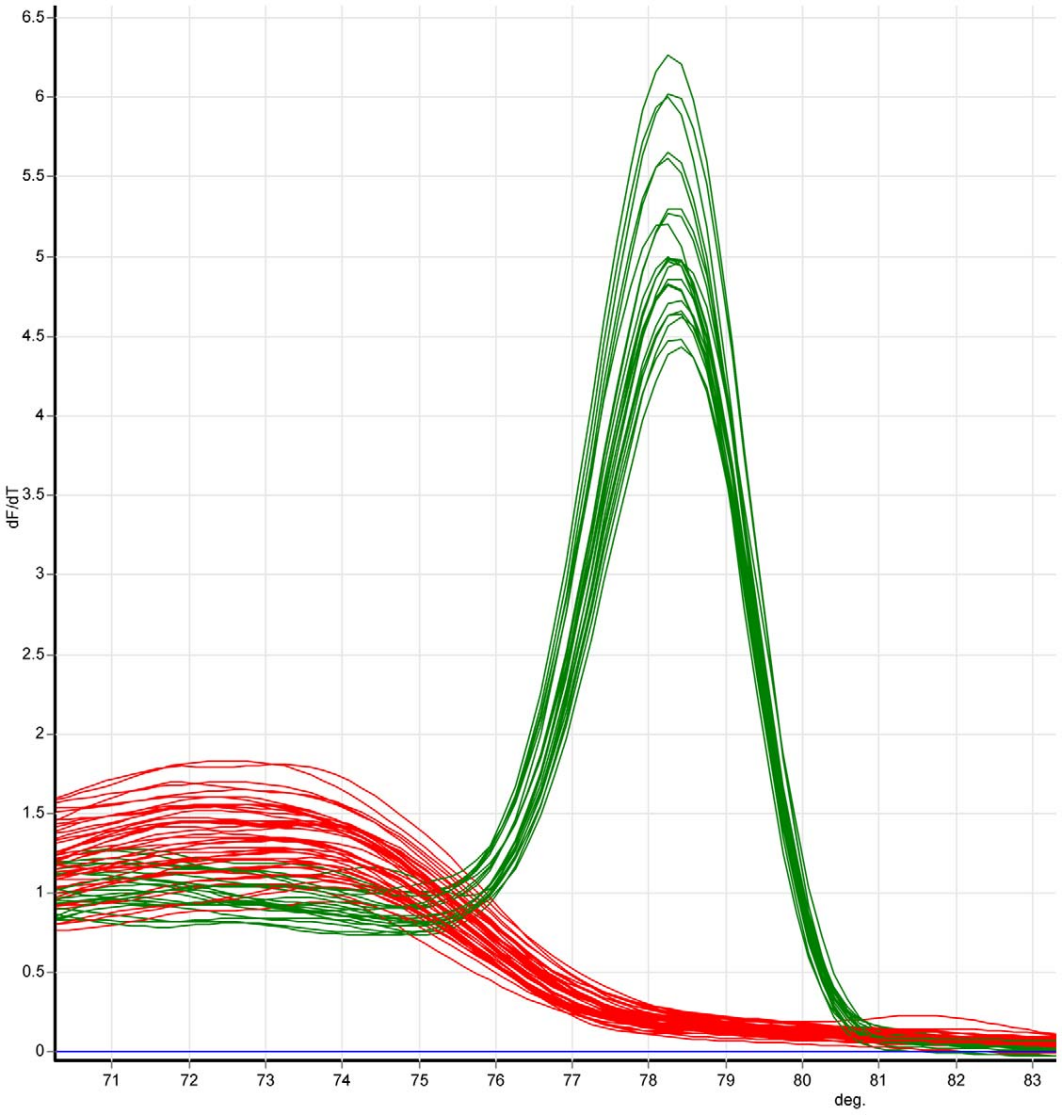
Marker typing in the BAC-range panel by MT-PCR-HRM. Each individual marker, which is uniquely amplified from genomic DNA, is mapped by genotyping in the mapping panel of 88 aliquots (Fig. 1b,f). This MT-PCR-HRM typing identifies the aliquots within the panel that are positive (green) or negative (red) for a particular marker by melting curve analysis using a fluorescent dye. If the PCR product is present in aliquots, the ‘positive’ green trace is detected and scored as 1. The absence of PCR product in aliquot (red line) is scored as 0. With a series of 1 and 0 for all aliquots in the panel, the marker is scored.

Multiplex Tandem (MT) and High Resolution Melting (HRM) PCR was used to ensure reliable amplification of STS markers from sub-genome template concentrations. This technique first requires a multiplex partial pre-amplification step of the panel with external primers, typically for only 20 thermo-cycles, so that PCR amplification is arrested in the log-linear phase and template concentration ratios are broadly retained [22], [23]. Importantly, this stage is highly amenable for multiplexing (up to 50 loci). The value of MT-PCR lies in its ability to reliably amplify from low copy templates within mixed samples when using BAC samples directly pooled from glycerol stocks for PCR. After dilution of the pre-amplification product, selective amplification and product identity is checked by PCR-HRM for each locus separately, this time using internal primers specific for individual STS markers. Thus, nested primers have to be designed for all STS markers. The MT-PCR-HRM technique is highly suitable for BAC pool screening for single copy targets and opens the possibility of direct screening from glycerol stocks with high speed, at low cost and with a minimum risk of false positive/negative results in a gel-free environment. The presence and identity of single copy STS markers within BAC pools is confirmed by diagnostic melt profiles following amplification (Figure 2 shows a first order differential plot of a melt profile).

### BAC Happy Mapping in Comparison with Sequence Data

We demonstrate the value of BAP mapping for merging linkage and physical maps into one pipeline re-using 40 out of 107 markers from the FCA region of *A. thaliana* (that had been previously ordered by conventional HAPPY mapping, [24]) for BAP mapping. First, the *A. thaliana* BAC library named PAC (Mi/P1) [25] containing twenty four 384-well plates was pooled in a 3D manner (Figure 1a) to create a BAC-range panel of 88 aliquots each containing ∼0.7 fold coverage of the genome. To minimize the risk of false negative results arising from amplification failure due to low concentration of target sequence within the BAC samples pooled from glycerol stocks, all 40 markers were subjected simultaneously to a multiplex PCR with external primers. In a second step, PCR-HRM is carried out separately for each individual marker, using primers targeting internal sites of the marker in a sample diluted from the first step (Figure 2).

**Figure 3:**
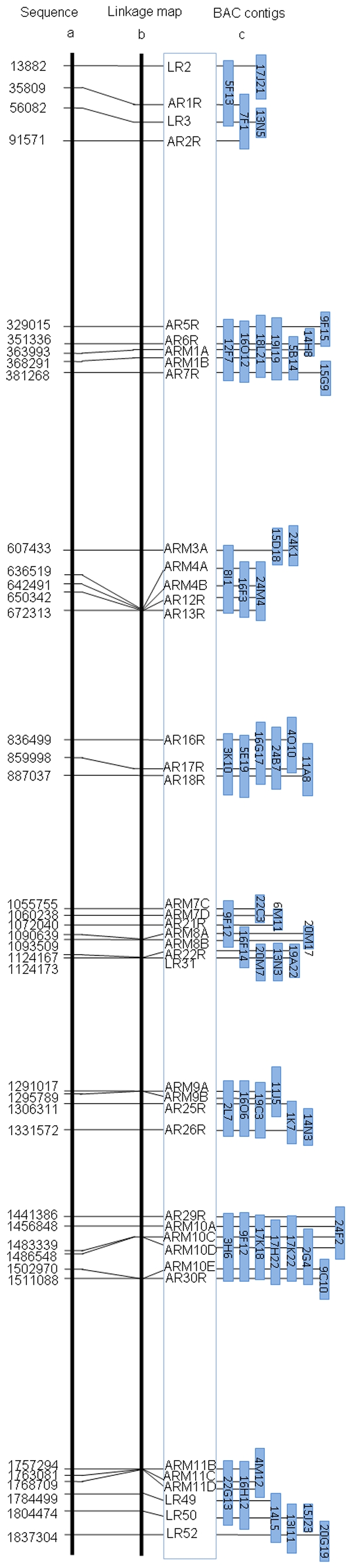
The physical map of FCA locus in comparison to its sequence. The sequence position indicated by the first nucleotide of the 40 markers belonging to the FCA locus (a) is reflected by the physical map (b – enframed and c) after BAP mapping. With the BAC range panel, the BACs (blue rectangles) harbouring the 40 markers are sorted into 8 contigs (c) and assembled by means of the long-range panel into a single linkage map (b) spanning the entire region of 1.8 Mbp.

Analysis of all mapping data showed that the mean DNA presentation of the BAC range-panel was 0.67x genomes per aliquot (GPA). The proportion and pattern of aliquots that are positive or negative for all markers reflected the relative assortment of these markers. Eight small linkage groups together containing positive BAC IDs for each typed marker were generated at a very stringent threshold of LOD = 6.0 (Figure 3). The BAC-range panel of ∼80 Kbp DNA inserts revealed linkage over distances of up to ∼65 Kbp (e.g. distance between ARM3A-AR13R), and correctly resolved markers spaced more than 4 Kbp (e.g. distance between ARM1A-ARM1B, ARM7C-ARM7D).

If two adjacent markers are more distant from each other than the size of the BAC clones (∼80 Kbp), they will segregate independently. Since the BAC library [25] was constructed based on partial digestion with restriction enzymes, possible non-random coverage of the genome is an additional explanation for gaps remaining in the map. Therefore, the long-range panel of genomic DNA was used for detecting linkage exceeding distances accommodated by the BAC range pools and for bridging missing BACs. Reference to genome sequence data revealed that the residual gaps between short contigs range from 110 Kbp (between AR26R-AR29R) to 241 Kbp (between AR30R-ARM11A), with most gaps larger than 160 Kbp (237 Kbp gap between AR2R-AR5R; 226 kb gap between AR7R-ARM3A; 164 Kbp gap between AR13R-AR16R; 169 Kbp gap between AR18R-ARM7C; 167 Kbp gap between LR31-ARM9A; Figure 3). The long range genomic DNA panel prepared by irradiation-induced breakage of embedded nuclei was constructed to correctly join the contigs into one linkage map. Markers chosen from the ends of the contigs of the BAC-range HAPPY/physical map (Figure 3) were typed in the ∼500 Kbp long-range DNA panel, thereby closing the gaps and allowing long-range ordering of markers. Using the long-range panel, the 8 small linkage groups could be joined into one physical map of 1.8 Mbp for the FCA region. The order of 40 markers was in perfect agreement with the sequencing data (Figure 3), even where the inter-marker spacing was only a few kilobases (e.g. distance between AR6R-ARM1A; ARM1A-ARM1B). Note that the use of a BAC library with x 5 genome coverage created the expected 2–7x redundancy in the number of BACs containing each marker (Figure 3).

### BAC Landing Data Transfer by Virtual Physical Mapping

One motivation behind our development and evaluation of BAP mapping was to convert existing BAC landing data into physical linkage mapping data. Integrating BAC landing data into the BAP mapping concept offers several advantages: (i) For species with large genomes, it is easier and faster to land STS markers by screening a BAC library than to work with a BAC-range panel. For instance, 290 to 300 384-well plates of a genomic BAC library with 100 kb average insert size are needed to be pooled together to get aliquots of 0.7 fold of the 16,000 Mbp wheat genome. It could be difficult to get reliable amplification of STS markers when using such BACs directly pooled from glycerol stocks due to excessive dilution of the template sequence(s) by *E. coli* DNA. (ii) By converting the BAC landing data into a physical linkage map, already existing sources of mapped STS markers may be used to accelerate BAP mapping. Conversion of the existing BAC landing data into physical linkage map bears the potential to support ongoing genome physical mapping and sequencing projects (e.g., for barley (http://barleygenome.org/), wheat (http://www.wheatgenome.org/), potato (http://www.potatogenome.net/), etc…) by accelerating the joining of small contigs. Based on the list of positive BAC IDs for 40 STS markers (Figure 3), we generated *de novo* a physical linkage map for these markers by using the BAP mapping concept: The *Arabidopsis* BAC library was randomly pooled by a computer to generate a virtual BAC-range panel with aliquots containing 0.7x genome coverage. The aliquots containing any BAC ID positive for an STS marker are scored as positive ( = 1). The aliquots not containing any positive BAC clone are scored as negative ( = 0). Analysis of the scoring results yielded 8 BAC linkage contigs, each with a marker order fitting perfectly to the previous BAP mapping data.

## Discussion

We have demonstrated that BAP mapping is a viable strategy for merging linkage and physical maps into one pipeline. The approach has several advantages over other mapping methods (Table 1).

**Table 1:** Comparison of BAP mapping and conventional HAPPY mapping.

	Happy mapping	BAC HAPPY MAPPING (BAP mapping)	Advantages of BAP over conventional HAPPY mapping
Principle	Utilizes a panel of High Molecular Weight nuclear DNA samples, each containing less than a full genome (typically 0.7x genome equivalents). A map is generated based on the premise that linked pairs of single copy STS markers will co-amplify in each sample significantly more frequently than unlinked markers, and in a manner that is proportional to their physical proximity.	BAP mapping combines conventional HAPPY mapping panels with similar panels created from pools of BAC clones (also representing 0.7x genome equivalents). Maps generated by the BAC panel provide fine resolution of marker order but is unable to span large gaps between contigs. Contig order is then resolved by mapping using the HAPPY map panel.	BAP mapping provides finer resolution of marker order than is generally possible by HAPPY mappingBAP mapping allows BAC landing, contig assembly and physical mapping to occur simultaneouslyBAC map panels can be recreated using the same clones. This means that ‘standard panels’ can be created for an organism or for particular initiativesBAP mapping allows users to utilize existing BAC resourcesStrict size selection of BACs is possible in BAP mappng. This provide finer control of map resolution. This may be of particular value for regions of intense interest.BAP mapping can yield a high resolution physical map (kbp resolution within Mbp range) even for chromosome regions of suppressed recombination and for large genomes.BAP mapping is the first strategy that merges genetic and physical mapping efforts into one pipeline, saving >50% of time, cost and efforts required for a physical mapping project
Short range mapping panel	A short-range panel is generated from nuclear DNA sheared to a size range of 50–300 kb.	A BAC library is pooled to create a panel with a homogeneous size range of ∼80–150 Kb according the average insert size of the BACs. Finer size selection is also possible.	It is easier and more precise to get aliquots containing 0.7-fold coverage of the genome by direct calculation rather than by empirical dilutionBAC panels ensure a higher and more precise resolution of the map than sheared DNA, and yield in addition a BAC tiling path from the linkage HAPPY map.
Long range mapping panels	A long-range panel is created from DNA of isolated nuclei sheared by irradiation to approximately 1–3 Mbp.	A long-range panel is created from DNA of isolated nuclei sheared by irradiation to 1–3 Mbp.	Both methods include a Mb-sized genomic DNA panel having the capability to effectively bridge ∼2 Mbp gaps.
Marker typing	PCR to detect the presence or absence of any single marker within the different aliquots on gels.	Multiplex tandem-PCR-High-Resolution-Melt analysis for marker typing.	MT-PCR-HRM analysis is a simple, rapid, gel-free, low cost and reliable means to amplify single copy targets from large BAC pools created directly from freezing stocks for up to 3 markers within a single reaction.
Output	Linkage HAPPY map	A strict physically integrated linkage map of high resolution and a BAC tiling path.	While a HAPPY map can only be used as skeleton guiding whole genome shotgun sequence (WGS) assembly, a BAP-map can guide sequence assembly from BAC by BAC sequence data as well as from WGS; it can also anchor BAC end derived markers to physical maps.The Possibility to convert existing BAC landing data into a physical linkage map bears the potential to support ongoing genome physical mapping and sequencing projects.The ability to overcome mapping and assembly problems raising from homogeneous high copy repeat blocks of up to 2 Mbp makes BAP mapping potentially important for physical mapping and sequencing of large genomes.

Mimicking recombination between genetic loci by creating artificial recombination points, BAP mapping has the potential to yield a high resolution physical map for entire chromosomes, without the limitation of insufficient supply of polymorphic loci. This strategy enables one to make a physical linkage map and BAC contigs even for chromosome regions of suppressed recombination.

Unlike conventional mapping technologies that generate physical maps by anchoring genetic maps to contig maps through BAC landing [11], BAP mapping does not require any pre-existing skeleton map. Application of the 3D BAC-range panel is particularly important when typing STS markers for linkage map generation, because the BAC clones containing the markers are identified simultaneously. Additionally, by using a BAC-range panel, a physical linkage map of very high resolution can be generated. It is likely that the map can precisely resolve markers spaced by only a few kilobase pairs (∼4 Kbp distances between ARM1A-ARM1B, Figure 3). Moreover, several markers (ARM1A-ARM1B, ARM7C-ARM7D, ARM10A-ARM10C-ARM10D, ARM11B-LR52) that were misplaced in a previous linkage map of the FCA locus [24], were correctly positioned by BAP mapping.

In addition to a high resolution based on the average BAC insert size, the BAP mapping method has the advantage of generating extended maps as the Mb-sized genomic DNA panel can bridge the gaps between BAC contigs. This ability is important for physical mapping and sequencing projects especially for large genomes where numerous blocks of highly repetitive sequences cause gaps in the maps due to the absence of unique markers. By mapping markers at both ends of the short linkage contigs, the order and orientation of these contigs are rapidly assembled in the long range physical linkage maps. In our study, the long range DNA panel of ∼500 Kbp was sufficient to bridge and assemble 8 BAC-range linkage contigs into a complete 1.8 Mbp physical linkage map with high resolution. Previous work using HAPPY mapping [17] has shown that the size of long range DNA panel can reach up to 2.5–3 Mbp. This means that the maximum distance between two adjacent markers that can be linked by means of a long range panel is ∼1–2 Mbp. Using three mapping panels of short-range, mid- and long-range, containing genomic fragments of 1.5, 2 and >2.5 Mbp, respectively, a HAPPY map consisting of 1001 STS markers spanning the entire 90 Mbp long arm of human chromosome 14 was constructed with a resolution of ∼100 Kbp [17]. This demonstrates that long range DNA panels provide an effective scaffold to build genome maps as well as to assemble the sequence contigs. Whereas present physical mapping and sequencing technologies are limited as to the long range assembly, BAP mapping could overcome this problem and might guide sequencing by merging genetic mapping, physical mapping and sequencing into a single pipeline. In the illustrative experiments described here, we limited scope of the study to 40 markers of known position along one linkage group (LG4) of *Arabidopsis thaliana*, although the application of more markers to the same pools could equally be used (or re-used) to generate genome-wide and/or more intensive genome coverage. Thus, we have demonstrated that BAP mapping is a simple technology with universal applications. A physical linkage map of Mbp range can be constructed with a resolution in the Kbp range and the capability to bridge ∼2 Mbp gaps that might arise by tandem repeats or other un-cloneable genomic sequences. The flexibility of the BAP mapping allows conversion of BAC landing data into BAC linkage contigs, utilizable for ongoing physical mapping and sequencing projects, especially for organisms with a large genome size. The 1.8 Mbp high resolution physical linkage map reported here for *A. thaliana* chromosome 4 demonstrated the potential of BAP mapping as a powerful tool in genomic map construction and sequence assembly.

## Methods

### Preparation of BAC-Range Panel

We used the Arabidopsis BAC library Mi/P1 [25] in this protocol as an example to demonstrate the usefulness of our BAP-mapping approach. The BAC library was carefully handled in flow cabinet to avoid cross-contamination. BACs were then pooled in a 3D manner (Figure 1a) to create BAC-range panels consisting of 88 aliquots, each containing about 0.7 haploid genome equivalents of DNA.

### Isolation of Nuclei from Leaf Tissue

Nuclei were isolated from 4-week-old *A. thaliana* plants by grinding 2 g of leaf material into a fine powder in liquid nitrogen before transfering into 200 mL of ice-cold wash buffer (10 mM Tris base; 80 mM KCl; 0.1 M EDTA; 10 mM spermidine; 10 mM spermine; 2% (w/v) Polyvinylpyrrolidone-40; 0.5 M sucrose; 0.5% (v/v) Triton X-100; 0.15% (v/v) β-mercaptoethanol; pH 9.3) and stirring gently for 10 min on ice. The suspension was then filtered through three layers of Miracloth (Merck chemicals) to remove tissue debris. The suspension containing nuclei was then subjected to three successive rounds of centrifugation (1,800 g for 20 min at 4°C) in 40 mL ice-cold wash buffer. The pelleted nuclei were re-suspended in 1 mL of homogenization buffer (10 mM Tris base; 80 mM KCl; 0.1 M EDTA; 10 mM spermidine; 10 mM spermine; 0.5 M sucrose; pH 9.3) and warmed to 45°C for 5 min. Nuclei were then embedded into an equal volume (1 mL) of 1% w/v low melting-point agarose solution (45°C) in glass capillaries (100 mL Supracaps, 1.2 mm internal diameter; Scientific Laboratory Supplies) using a wide-bore pipette tip. The final concentration within the agarose strings was ∼10^6^ nuclei/mL. After 15 min at 4°C, the embedded agarose strings were transferred to 40 mL lysis buffer (0.5 M EDTA; 1% (w/v) sodium lauryl sarcosine; 0.1 mg/mL proteinase K; pH 9.3) and incubated for 48 h at 45°C under continuous gentle shaking (50 rpm). Strings were then washed in 50 mL EDTA (0.5 M, pH 9.0) for 1 h at 50°C; in 50 mL EDTA (0.05 M, pH 8.0) for 1 h on ice and finally 3 times in TE (Tris∶EDTA = 20 mM∶50 mM) for 1 h on ice. The washed strings were stored in TE (20∶50) at 4°C.

### Preparation of Long-Range Mapping Panels

An agarose string was γ-irradiated (35 J/kg; Gravatom RX30/55M 137Cs source) to randomly break the nuclear DNA, and loaded across a single well in a gel of 1% w/v chromosomal grade agarose (Bio-Rad) in 0.5x TBE, together with *Saccharomyces cerevisiae* chromosomal markers (Bio-Rad), and subjected to pulsed-field gel electrophoresis (180 V, 100-s pulse time, 16 h). After electrophoresis, the gel segments containing the chromosomal markers was excised and stained with ethidium bromide to visualize DNA under UV light. The central gel portion was incubated for 3 h in 375 mL 0.1xTE at 4°C (TE buffer re-newed every hour). After removing the gel from 0.1x TE, a glass capillary (internal diameter 0.48 mm; Drummond Scientific Co.) was used to remove agarose plugs from the gel region in the size range of ∼500 Kbp, indicated by the *S. cerevisiae* markers. One plug was transferred into each well of a 96-well PCR plate (Thermowell, Costar) and overlaid with ∼30 µl of light mineral oil (Sigma).

### Panel Evaluation and Marker Typing

Before typing all markers, the DNA content of the mapping panel needed to be checked to make sure that each well harbours 0.5–0.7 genomes per aliquot (GPA). This calculation was based on the analysis of the first few markers using the formula GPA = –log_e_([T-N]/*T*), where *T* is the total number of aliquots analysed, and *N* is the number of aliquots positive for the marker (PanelStats software, PHD, unpublished).

We used three steps for marker typing: a whole genome PCR amplification, a multiplexed pre-amplification and hemi-nested marker-specific amplification. The whole genome amplification of the panel consists of a primer extension pre-amplification (PEP) with a random 15-mer primer [26]. The reaction mix (10 µL) contained of 1x Amplitaq Buffer II (PE Applied Biosystems), 2.5 mM MgCl_2_, 200 µM of each dNTP, 10 µM N15 primer (Operon Technologies, Inc., Alameda CA), 1 U *Taq* polymerase (AmpliTaq, PE Applied Biosystems) in addition to the agarose plugs. The PCR cycling conditions used were: 93°C for 5 min; then 50 cycles of 94°C for 30 s, 37°C for 2 min, 37–55°C ramp over 3 min, and 55°C for 4 min. PEP products were then diluted to 150 µL in HPLC-grade water and stored at 70°C until needed as templates for multiplexed pre-amplification.

The multiplex marker pre-amplification reaction (step2) contained 5 µL of the diluted PEP product, 0.25 µM of each primer (forward-external, forward internal and reverse external for each of up to 50 markers), 1x AmpliTaq Gold reaction buffer, 1 unit AmpliTaq Gold polymerase (PE Applied Biosystems), 4 mM MgCl_2_ and 200 µM of each dNTP. PCR cycling conditions used were: 93°C for 10 min; then 22 cycles of 94°C for 20 s, 55°C for 30 s and 72°C for 1 min. Amplification products were diluted in HPLC grade water to a final volume of 250 µL and stored at −20°C.

Individual markers are then typed by ‘Multiplex PCR and High Resolution Melt’ (MT-PCR-HRM, step 3) in 10 µl reactions consisting of 2 µL of the diluted pre-amplification product, 5 µl of SensiMix*Plus* SYBR (Quantace) and 5 µM of each forward and reverse primer. The MT-PCR-HRM was performed in a Rotor-Gene 6000 (Qiagen, UK) using the following conditions: 95°C for 10 min, followed by 35 cycles at 95°C for 20 s, 57°C for 30 s and 72°C for 50 s; the detection option ‘green’ was used to monitor fluorescence during each cycle at 72°C. High resolution melting analysis was performed at a ramp from 65°C to 90°C, increasing by 0.3°C each step, holding for 90 s prior to each melt, and also holding for 2 s after each melt.

### Data Analysis

Pairwise LOD scores, calculated using the Lodulator program, are entered in the Lontig program to attribute the markers to linkage groups [16], [17], [19]. The optimal order and spacing of markers for each linkage group was determined using the *DGmap* software [27].
